# Supplementary material for: Does membrane feeding compromise the quality of Aedes aegypti mosquitoes?
Source: PLoS One. 2019 Nov 6;14(11):e0224268. doi: 10.1371/journal.pone.0224268 (PMC6834243; doi:10.1371/journal.pone.0224268)
Supplement: S1 Appendix — (DOCX) [file pone.0224268.s002.docx]

**S1 Appendix.** **General linear models for all parameters tested in the study.**

**Female wing length**

| Source | | Type III Sum of Squares | df | Mean Square | F | P |
| --- | --- | --- | --- | --- | --- | --- |
| Intercept | Hypothesis | 1246.041 | 1 | 1246.041 | 38520.624 | **< 0.0001** |
|  | Error | .130 | 4.003 | .032^a^ |  |  |
| Population origin | Hypothesis | .002 | 1 | .002 | 0.070 | 0.804 |
|  | Error | .130 | 4.003 | .032^b^ |  |  |
| Blood source | Hypothesis | .040 | 1 | .040 | 1.248 | 0.326 |
|  | Error | .130 | 4.003 | .032^c^ |  |  |
| Replicate population (Population origin * Blood source) | Hypothesis | .130 | 4 | .032 | 3.387 | **0.011** |
|  | Error | 1.300 | 136 | .010^d^ |  |  |
| Population origin * Blood source | Hypothesis | .006 | 1 | .006 | .199 | 0.678 |
|  | Error | .130 | 4.003 | .032^a^ |  |  |

**Male wing length**

| Source | | Type III Sum of Squares | df | Mean Square | F | P |
| --- | --- | --- | --- | --- | --- | --- |
| Intercept | Hypothesis | 697.849 | 1 | 697.849 | 43445.866 | **< 0.0001** |
|  | Error | .064 | 4.002 | .016^a^ |  |  |
| Population origin | Hypothesis | .006 | 1 | .006 | 0.355 | 0.584 |
|  | Error | .064 | 4.002 | .016^b^ |  |  |
| Blood source | Hypothesis | .058 | 1 | .058 | 3.630 | 0.129 |
|  | Error | .064 | 4.002 | .016^c^ |  |  |
| Replicate population (Population origin * Blood source) | Hypothesis | .064 | 4 | .016 | 4.298 | **0.003** |
|  | Error | .501 | 134 | .004^d^ |  |  |
| Population origin * Blood source | Hypothesis | .002 | 1 | .002 | 0.133 | 0.734 |
|  | Error | .064 | 4.002 | .016^c^ |  |  |

**Female development time**

| Source | | Type III Sum of Squares | df | Mean Square | F | P |
| --- | --- | --- | --- | --- | --- | --- |
| Intercept | Hypothesis | 1888.657 | 1 | 1888.657 | 81382.730 | **< 0.0001** |
|  | Error | .093 | 4.010 | .023^a^ |  |  |
| Population origin | Hypothesis | .012 | 1 | .012 | 0.496 | 0.520 |
|  | Error | .093 | 4.010 | .023^a^ |  |  |
| Blood source | Hypothesis | .442 | 1 | .442 | 19.064 | **0.012** |
|  | Error | .093 | 4.010 | .023^b^ |  |  |
| Replicate population (Population origin * Blood source) | Hypothesis | .093 | 4 | .023 | 1.308 | 0.284 |
|  | Error | .692 | 39 | .018^c^ |  |  |
| Population origin * Blood source | Hypothesis | .255 | 1 | .255 | 10.985 | **0.029** |
|  | Error | .093 | 4.010 | .023^d^ |  |  |

**Male development time**

| Source | | Type III Sum of Squares | df | Mean Square | F | P |
| --- | --- | --- | --- | --- | --- | --- |
| Intercept | Hypothesis | 1695.010 | 1 | 1695.010 | 82788.477 | **< 0.0001** |
|  | Error | .082 | 4.006 | .020^a^ |  |  |
| Population origin | Hypothesis | .039 | 1 | .039 | 1.882 | 0.242 |
|  | Error | .082 | 4.006 | .020^a^ |  |  |
| Blood source | Hypothesis | .625 | 1 | .625 | 30.510 | **0.005** |
|  | Error | .082 | 4.006 | .020^b^ |  |  |
| Replicate population (Population origin * blood source) | Hypothesis | .082 | 4 | .020 | 2.267 | 0.079 |
|  | Error | .353 | 39 | .009^c^ |  |  |
| Population origin * Blood source | Hypothesis | .183 | 1 | .183 | 8.955 | **0.040** |
|  | Error | .082 | 4.006 | .020^d^ |  |  |

**Survival to adulthood**

| Source | | Type III Sum of Squares | df | Mean Square | F | P |
| --- | --- | --- | --- | --- | --- | --- |
| Intercept | Hypothesis | 98.519 | 1 | 98.519 | 14522.464 | **< 0.0001** |
|  | Error | .027 | 4.009 | .007^a^ |  |  |
| Population origin | Hypothesis | .011 | 1 | .011 | 1.660 | 0.267 |
|  | Error | .027 | 4.009 | .007^a^ |  |  |
| Blood source | Hypothesis | .041 | 1 | .041 | 6.044 | 0.070 |
|  | Error | .027 | 4.009 | .007^b^ |  |  |
| Replicate population (Population origin * blood source) | Hypothesis | .027 | 4 | .007 | 1.462 | 0.232 |
|  | Error | .181 | 39 | .005^c^ |  |  |
| Population origin * Blood source | Hypothesis | .005 | 1 | .005 | 0.785 | 0.425 |
|  | Error | .027 | 4.009 | .007^d^ |  |  |

**Fecundity on human arm**

| Source | | Type III Sum of Squares | df | Mean Square | F | P |
| --- | --- | --- | --- | --- | --- | --- |
| Intercept | Hypothesis | 791492.379 | 1 | 791492.379 | 6935.428 | **< 0.0001** |
|  | Error | 461.549 | 4.044 | 114.123^a^ |  |  |
| Population origin | Hypothesis | 105.948 | 1 | 105.948 | 0.928 | 0.389 |
|  | Error | 461.549 | 4.044 | 114.123^b^ |  |  |
| Blood source | Hypothesis | 4269.309 | 1 | 4269.309 | 37.410 | **0.003** |
|  | Error | 461.549 | 4.044 | 114.123^b^ |  |  |
| Replicate population (Population origin * blood source) | Hypothesis | 454.707 | 4 | 113.677 | 0.288 | 0.885 |
|  | Error | 48960.616 | 124 | 394.844^c^ |  |  |
| Population origin * Blood source | Hypothesis | 3112.234 | 1 | 3112.234 | 27.271 | **0.006** |
|  | Error | 461.549 | 4.044 | 114.123^b^ |  |  |

**Fecundity on membrane feeder**

| Source | | Type III Sum of Squares | df | Mean Square | F | P |
| --- | --- | --- | --- | --- | --- | --- |
| Intercept | Hypothesis | 606060.674 | 1 | 606060.674 | 2206.385 | **< 0.0001** |
|  | Error | 1103.942 | 4.019 | 274.685^a^ |  |  |
| Population origin | Hypothesis | 1912.486 | 1 | 1912.486 | 6.962 | 0.057 |
|  | Error | 1103.942 | 4.019 | 274.685^b^ |  |  |
| Blood source | Hypothesis | 11.279 | 1 | 11.279 | 0.041 | 0.849 |
|  | Error | 1103.942 | 4.019 | 274.685^c^ |  |  |
| Replicate population (Population origin * blood source) | Hypothesis | 1097.766 | 4 | 274.441 | 0.624 | 0.646 |
|  | Error | 59346.591 | 135 | 439.604^d^ |  |  |
| Population origin * Blood source | Hypothesis | 1101.538 | 1 | 1101.538 | 4.010 | 0.115 |
|  | Error | 1103.942 | 4.019 | 274.685^b^ |  |  |

**Fecundity on both blood sources**

| Source | | Type III Sum of Squares | df | Mean Square | F | P |
| --- | --- | --- | --- | --- | --- | --- |
| Intercept | Hypothesis | 1397702.542 | 1 | 1397702.542 | 1599.684 | **< 0.0001** |
|  | Error | 4380.757 | 5.014 | 873.737^a^ |  |  |
| Population origin | Hypothesis | 1525.593 | 1 | 1525.593 | 1.745 | 0.244 |
|  | Error | 4379.187 | 5.010 | 874.120^b^ |  |  |
| Blood source selected on | Hypothesis | 2088.797 | 1 | 2088.797 | 2.391 | 0.183 |
|  | Error | 4380.757 | 5.014 | 873.737^c^ |  |  |
| Blood source fed on | Hypothesis | 10771.667 | 1 | 10771.667 | 25.897 | **< 0.0001** |
|  | Error | 110225.979 | 265 | 415.947^d^ |  |  |
| Replicate population (Population origin * blood source) | Hypothesis | 4375.333 | 5 | 875.067 | 2.104 | 0.065 |
|  | Error | 110225.979 | 265 | 415.947^d^ |  |  |
| Blood source selected on * Blood source fed on | Hypothesis | 2363.294 | 1 | 2363.294 | 5.682 | **0.018** |
|  | Error | 110225.979 | 265 | 415.947^d^ |  |  |

**Hatch proportion on human arm**

| Source | | Type III Sum of Squares | df | Mean Square | F | P |
| --- | --- | --- | --- | --- | --- | --- |
| Intercept | Hypothesis | 228.403 | 1 | 228.403 | 1594.540 | **< 0.0001** |
|  | Error | 0.574 | 4.004 | .143^a^ |  |  |
| Population origin | Hypothesis | 0.161 | 1 | 0.161 | 1.127 | 0.348 |
|  | Error | 0.574 | 4.004 | .143^b^ |  |  |
| Blood source | Hypothesis | 0.780 | 1 | 0.780 | 5.445 | 0.080 |
|  | Error | 0.574 | 4.004 | .143^b^ |  |  |
| Replicate population (Population origin * blood source) | Hypothesis | 0.574 | 4 | 0.143 | 2.833 | **0.027** |
|  | Error | 6.275 | 124 | .051^c^ |  |  |
| Population origin * Blood source | Hypothesis | 0.083 | 1 | 0.083 | 0.580 | 0.489 |
|  | Error | 0.574 | 4.004 | .143^b^ |  |  |

**Hatch proportion on membrane feeder**

| Source | | Type III Sum of Squares | df | Mean Square | F | P |
| --- | --- | --- | --- | --- | --- | --- |
| Intercept | Hypothesis | 249.812 | 1 | 249.812 | 7489.095 | **< 0.0001** |
|  | Error | 0.134 | 4.022 | .033^a^ |  |  |
| Population origin | Hypothesis | 0.104 | 1 | 0.104 | 3.104 | 0.153 |
|  | Error | 0.134 | 4.022 | .033^b^ |  |  |
| Blood source | Hypothesis | 0.000 | 1 | 0.000 | 0.013 | 0.913 |
|  | Error | 0.134 | 4.022 | .033^c^ |  |  |
| Replicate population (Population origin * blood source) | Hypothesis | 0.133 | 4 | 0.033 | 0.537 | 0.708 |
|  | Error | 8.368 | 135 | .062^d^ |  |  |
| Population origin * Blood source | Hypothesis | 0.054 | 1 | 0.054 | 1.619 | 0.272 |
|  | Error | 0.134 | 4.022 | .033^b^ |  |  |

**Hatch proportion on both blood sources**

| Source | | Type III Sum of Squares | df | Mean Square | F | P |
| --- | --- | --- | --- | --- | --- | --- |
| Intercept | Hypothesis | 478.343 | 1 | 478.343 | 3988.700 | **< 0.0001** |
|  | Error | .601 | 5.014 | .120^a^ |  |  |
| Population origin | Hypothesis | .261 | 1 | .261 | 2.172 | 0.200 |
|  | Error | .601 | 5.010 | .120^b^ |  |  |
| Blood source selected on | Hypothesis | .426 | 1 | .426 | 3.552 | 0.118 |
|  | Error | .601 | 5.014 | .120^c^ |  |  |
| Blood source fed on | Hypothesis | .001 | 1 | .001 | .010 | 0.919 |
|  | Error | 14.896 | 265 | .056^d^ |  |  |
| Replicate population (Population origin * blood source) | Hypothesis | .601 | 5 | .120 | 2.137 | 0.061 |
|  | Error | 14.896 | 265 | .056^d^ |  |  |
| Blood source selected on * Blood source fed on | Hypothesis | .398 | 1 | .398 | 7.088 | **0.008** |
|  | Error | 14.896 | 265 | .056^d^ |  |  |

**Blood meal weight**

| Source | | Type III Sum of Squares | df | Mean Square | F | P |
| --- | --- | --- | --- | --- | --- | --- |
| Intercept | Hypothesis | 1022.093 | 1 | 1022.093 | 792.931 | **<0.0001** |
|  | Error | 3.451 | 2.677 | 1.289^a^ |  |  |
| Population origin | Hypothesis | 2.175 | 1 | 2.175 | 1.610 | .273 |
|  | Error | 5.403 | 4 | 1.351^b^ |  |  |
| Blood source | Hypothesis | .215 | 1 | .215 | .159 | .710 |
|  | Error | 5.403 | 4 | 1.351^b^ |  |  |
| Replicate population (Population origin * blood source) | Hypothesis | 5.403 | 4 | 1.351 | 2.913 | **0.027** |
|  | Error | 32.919 | 71 | .464^c^ |  |  |
| Experiment date | Hypothesis | .402 | 1 | .402 | .867 | .355 |
|  | Error | 32.919 | 71 | .464^c^ |  |  |
| Population origin * Blood source | Hypothesis | .063 | 1 | .063 | .047 | .839 |
|  | Error | 5.403 | 4 | 1.351^b^ |  |  |

**(log) Feeding duration**

| Source | | Type III Sum of Squares | df | Mean Square | F | P |
| --- | --- | --- | --- | --- | --- | --- |
| Intercept | Hypothesis | 519.083 | 1 | 519.083 | 4509.327 | **0.001** |
|  | Error | .194 | 1.685 | .115^a^ |  |  |
| Population origin | Hypothesis | .004 | 1 | .004 | 0.498 | 0.519 |
|  | Error | .035 | 4 | .009^b^ |  |  |
| Blood source | Hypothesis | .012 | 1 | .012 | 1.344 | 0.311 |
|  | Error | .035 | 4 | .009^b^ |  |  |
| Replicate population (Population origin * blood source) | Hypothesis | .035 | 4 | .009 | 0.466 | 0.760 |
|  | Error | 2.085 | 110 | .019^c^ |  |  |
| Experiment date | Hypothesis | .250 | 2 | .125 | 6.606 | **0.002** |
|  | Error | 2.085 | 110 | .019^c^ |  |  |
| Population origin * Blood source | Hypothesis | .010 | 1 | .010 | 1.177 | 0.339 |
|  | Error | .035 | 4 | .009^b^ |  |  |

**(log) Host-seeking duration in laboratory cage**

| Source | | Type III Sum of Squares | df | Mean Square | F | P |
| --- | --- | --- | --- | --- | --- | --- |
| Intercept | Hypothesis | 587.141 | 1 | 587.141 | 5939.986 | **< 0.0001** |
|  | Error | .398 | 4.025 | .099^a^ |  |  |
| Population origin | Hypothesis | .106 | 1 | .106 | 1.069 | 0.359 |
|  | Error | .398 | 4.025 | .099^b^ |  |  |
| Blood source | Hypothesis | .086 | 1 | .086 | .865 | 0.405 |
|  | Error | .398 | 4.025 | .099^c^ |  |  |
| Replicate population (Population origin * blood source) | Hypothesis | .395 | 4 | .099 | .505 | 0.732 |
|  | Error | 52.362 | 268 | .195^d^ |  |  |
| Population origin * Blood source | Hypothesis | .325 | 1 | .325 | 3.288 | 0.144 |
|  | Error | .398 | 4.025 | .099^c^ |  |  |

**Proportion feeding on human arms**

| Source | | Type III Sum of Squares | df | Mean Square | F | P |
| --- | --- | --- | --- | --- | --- | --- |
| Intercept | Hypothesis | 47.958 | 1 | 47.958 | 822.265 | **< 0.001** |
|  | Error | .135 | 2.322 | .058^a^ |  |  |
| Population origin | Hypothesis | .004 | 1 | .004 | 0.237 | .652 |
|  | Error | .064 | 4 | .016^b^ |  |  |
| Blood source | Hypothesis | .025 | 1 | .025 | 1.583 | .277 |
|  | Error | .064 | 4 | .016^b^ |  |  |
| Experiment date | Hypothesis | .106 | 2 | .053 | 5.079 | **.022** |
|  | Error | .146 | 14 | .010^c^ |  |  |
| Replicate population (Population origin * blood source) | Hypothesis | .064 | 4 | .016 | 1.531 | .247 |
|  | Error | .146 | 14 | .010^c^ |  |  |
| Population origin * Blood source | Hypothesis | .018 | 1 | .018 | 1.115 | .351 |
|  | Error | .064 | 4 | .016^b^ |  |  |

**Proportion feeding on membrane feeders**

| Source | | Type III Sum of Squares | df | Mean Square | F | P |
| --- | --- | --- | --- | --- | --- | --- |
| Intercept | Hypothesis | 4.485 | 1 | 4.485 | 10.396 | 0.101 |
|  | Error | .747 | 1.732 | .431^a^ |  |  |
| Population origin | Hypothesis | .016 | 1 | .016 | 1.175 | 0.339 |
|  | Error | .055 | 4 | .014^b^ |  |  |
| Blood source | Hypothesis | .245 | 1 | .245 | 17.753 | **0.014** |
|  | Error | .055 | 4 | .014^b^ |  |  |
| Experiment date | Hypothesis | .926 | 2 | .463 | 10.180 | **0.002** |
|  | Error | .637 | 14 | .045^c^ |  |  |
| Replicate population (Population origin * blood source) | Hypothesis | .055 | 4 | .014 | 0.303 | 0.871 |
|  | Error | .637 | 14 | .045^c^ |  |  |
| Population origin * Blood source | Hypothesis | .017 | 1 | .017 | 1.264 | 0.324 |
|  | Error | .055 | 4 | .014^b^ |  |  |

**Attraction to human hand in olfactometer**

| Source | | Type III Sum of Squares | df | Mean Square | F | P |
| --- | --- | --- | --- | --- | --- | --- |
| Intercept | Hypothesis | 6.139 | 1 | 6.139 | 805.067 | **< 0.0001** |
|  | Error | 0.030 | 4 | .008^a^ |  |  |
| Blood source | Hypothesis | 0.109 | 1 | 0.109 | 14.235 | **0.020** |
|  | Error | 0.030 | 4 | .008^a^ |  |  |
| Population origin | Hypothesis | 0.056 | 1 | 0.056 | 7.365 | 0.053 |
|  | Error | 0.030 | 4 | .008^a^ |  |  |
| Replicate population (Population origin * blood source) | Hypothesis | 0.030 | 4 | 0.008 | 0.259 | 0.901 |
|  | Error | 0.589 | 20 | .029^b^ |  |  |
| Population origin * Blood source | Hypothesis | 1.315E-05 | 1 | 1.315E-05 | 0.002 | 0.969 |
|  | Error | 0.030 | 4 | .008^a^ |  |  |

**Attraction to heat in olfactometer**

| Source | | Type III Sum of Squares | df | Mean Square | F | P |
| --- | --- | --- | --- | --- | --- | --- |
| Intercept | Hypothesis | 0.437 | 1 | 0.437 | 133.926 | **< 0.0001** |
|  | Error | 0.014 | 4.321 | .003^a^ |  |  |
| Blood source | Hypothesis | 0.023 | 1 | 0.023 | 7.127 | 0.052 |
|  | Error | 0.014 | 4.321 | .003^b^ |  |  |
| Population origin | Hypothesis | 0.002 | 1 | 0.002 | 0.526 | 0.506 |
|  | Error | 0.014 | 4.321 | .003^b^ |  |  |
| Replicate population (Population origin * blood source) | Hypothesis | 0.013 | 4 | 0.003 | 0.429 | 0.786 |
|  | Error | 0.179 | 24 | .007^c^ |  |  |
| Population origin * Blood source | Hypothesis | 0.003 | 1 | 0.003 | 0.916 | 0.389 |
|  | Error | 0.014 | 4.321 | .003^b^ |  |  |

**Attraction to human odor in olfactometer**

| Source | | Type III Sum of Squares | df | Mean Square | F | P |
| --- | --- | --- | --- | --- | --- | --- |
| Intercept | Hypothesis | 2.236 | 1 | 2.236 | 204.019 | **< 0.0001** |
|  | Error | 0.044 | 4.031 | .011^a^ |  |  |
| Blood source | Hypothesis | 0.021 | 1 | 0.021 | 1.887 | 0.241 |
|  | Error | 0.044 | 4.031 | .011^b^ |  |  |
| Population origin | Hypothesis | 0.003 | 1 | 0.003 | 0.273 | 0.629 |
|  | Error | 0.044 | 4.031 | .011^c^ |  |  |
| Replicate population (Population origin * blood source) | Hypothesis | 0.044 | 4 | 0.011 | 0.538 | 0.709 |
|  | Error | 0.549 | 27 | .020^d^ |  |  |
| Population origin * Blood source | Hypothesis | 0.007 | 1 | 0.007 | 0.648 | 0.466 |
|  | Error | 0.044 | 4.031 | .011^e^ |  |  |
